# A scarce dataset for ancient Arabic handwritten text recognition

**DOI:** 10.1016/j.dib.2024.110813

**Published:** 2024-08-08

**Authors:** Rayyan Najam, Safiullah Faizullah

**Affiliations:** Department of Computer Science, Islamic University, Madinah 42351, Saudi Arabia

**Keywords:** Optical character recognition, Arabic and ancient handwritten document analysis, Ancient Arabic OCR, Deep learning, Computer vision, Natural language processing, Text correction, Large language models

## Abstract

Developing Deep Learning Optical Character Recognition is an active area of research, where models based on deep neural networks are trained on data to eventually extract text within an image. Even though many advances are currently being made in this area in general, the Arabic OCR domain notably lacks a dataset for ancient manuscripts. Here, we fill this gap by providing both the image and textual ground truth for a collection of ancient Arabic manuscripts. This scarce dataset is collected from the central library of the Islamic University of Madinah, and it encompasses rich text spanning different geographies across centuries. Specifically, eight ancient books with a total of forty pages, both images and text, transcribed by the experts, are present in this dataset. Particularly, this dataset holds a significant value due to the unavailability of such data publicly, which conspicuously contributes to the deep learning models development/augmenting, validation, testing, and generalization by researchers and practitioners, both for the tasks of Arabic OCR and Arabic text correction.

Specifications Table

**Table:** 

Subject	Computer Science: Artificial Intelligence; Data Science: Applied Machine Learning
Specific subject area	Optical Character Recognition; Handwritten Text Recognition; Deep Learning; Computer Vision; Natural Language Processing; Large Language Model; Arabic OCR; Ancient Handwritten Document Analysis
Data format	Raw: PNG Analyzed: Text in Word Files
Type of data	Image, Text
Data collection	The dataset has been collected from the Central Library of the Islamic University of Madinah. Originally, it only consisted of scanned images of ancient manuscripts spanning different eras and places. Further, a team of expert librarians assisted in classifying fifty selected books, of which eight were selected, to ensure maximum diversity of fonts and quality of images. Images of low quality, poor handwriting, or poor page conditions were excluded. Next, five pages from each bucket were selected to be cropped in photo processing software, then given to specialists to annotate as text in text processing software.
Data source location	Institution: Central Library, Islamic University of Madinah City: Madinah Country: Saudi Arabia
Data accessibility	Repository name: Historical Arabic Handwritten Text Recognition Dataset Data identification number: 10.17632/xz6f8bw3w8.1 Direct URL to data: https://data.mendeley.com/datasets/xz6f8bw3w8/1
Related research article	R. Najam, S. Faizullah, Analysis of Recent Deep Learning Techniques for Arabic Handwritten-Text OCR and Post-OCR Correction, Applied Sciences 13 (2023) 7568. https://doi.org/10.3390/app13137568.

## 1 Value of the Data

• There is no publicly available dataset for Ancient Arabic Handwritten Text, along with the ground truth text, prior to this data, to our knowledge. This data fills this very significant gap in the dataset domain.
• This dataset, in its vision part, can be used in developing OCR solutions for Ancient Arabic Text. Furthermore, it can be intuitively used to enhance general Arabic OCR models. In addition to OCR, these images can be used for developing multiple document analysis solutions, such as analyzing manuscript dates, analyzing and identifying writers, and developing digitization pipelines [[1], [2], [3]].
• The textual part plays a crucial and significant role in the evaluation and testing of the OCR systems using different metrics of accuracy. The textual part, even without the vision part, is of significant importance, as it can be used to develop and evaluate Arabic text correction deep learning models, such as in [4], a domain that lacks such a historical dataset. Text correction models do not require character-level annotation, as the rectification result can be compared on a custom page or line level, both of which this dataset provides [5].
• A combination of the visual and textual data can be fed to Deep/Machine Learning models first to recognize the text present in the images, then correct the produced text. OCR models do not generally require character annotation. For example, in the training phase, Tesseract uses a provided corpus of text to generate a synthetic dataset used to train the model [6]. Moreover, for testing, the comparison is mainly between the ground truth and the resultant text from applying the OCR to the images [7]; therefore, this dataset can be utilized for both training and testing purposes. This can be applied to different architectures, such as Convolutional Neural Networks, Long Short-term Memory, Generative Adversarial Networks, and Transformer, where it is up to the user to adjust the data fed at a desired level of annotation or to use it for generating more data [8].
• Not only is this dataset limited to Arabic but it can also be used to develop and test same-script and multilingual OCR deep learning models. Multilingual models, such as Arabic with Urdu, use a variety of datasets coming from different languages, and this dataset can contribute to being a part representing the Arabic text, either entirely or partially by being a small part of a much larger Arabic dataset [9]. Similarly, a multilingual text correction model that includes ancient Arabic can predominantly benefit from this dataset to correct the text generated from OCR, both for current and old Arabic scripts [10].
• Researchers in Artificial Intelligence, Machine/Deep Learning, Natural Language Processing, Computer Vision, Large Language Models can primarily benefit from such rare data in developing and evaluating their models, whether as images only, text only, or a mixture of both. Further, historians, paleographers, epistemologists, humanitarians, social scientists, and linguists can use both types of data to promote studying and analyzing Arabic and Islamic literature, scripts, history, and society and gain remarkable insights either from the text, the ancient images, or the models they will be working with built to their use case.
• The focus groups that can use this data are mainly the Ancient Arabic and Arabic OCR or text correctionʼs deep learning practitioners and researchers. For them, the data will be beneficial in the following situations: using the dataset for testing ancient or ancient Arabic OCR models, whether purely on this dataset or on a mixture with other datasets; using the dataset for training ancient or ancient Arabic OCR models, especially with generative and augmentative techniques, and by combining them with other models; and using the dataset for testing or training Arabic or Ancient Arabic text correction models, whether incorporated with an existing model and data or purely on this data.

## 2 Background

Optical character recognition is the process of extracting the text present within an image. Typically, deep learning models are developed to perform such a task because of the capabilities they have to learn from data. These models mainly use deep neural networks, stacking multiple hidden layers to learn the latent representation from the data. In particular, datasets are used to develop various DL models and their combinations, such as Convolutional Neural Networks (CNNs) [11], Long Short-Term Memory (LSTMs) [12], and Transformer [13], and then evaluate them [14]. The majority of the data is used for training, while the test is done on a minor part or totally new unseen data. To our knowledge, although there are some datasets for printed and handwritten Arabic in general, such as KHATT [15], IFN/ENIT [16], HACDB [17], and AHDB [[18], [19]], the availability of a public dataset with ground truth text along with the ancient handwritten images for OCR remains rather none. Therefore, to fill this gap in the dataset domain, this dataset has been collected, curated, analyzed, transcribed, and evaluated by experts [20]. This dataset has been collected from various original old manuscripts present at the Central Library of the Islamic University of Madinah.

## 3 Data Description

**Fig. 1:**
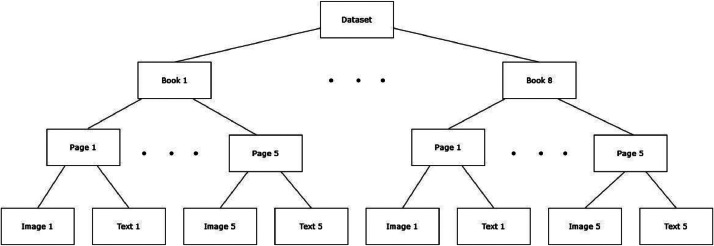
Structure of the dataset directory.

The dataset is organized in a way that allows all different researchers and practitioners to easily access the data they need, whether it's text or image. To elaborate on the description, initially, the library had scanned images of ancient manuscripts encompassing books from different eras and places. It was not easy to select a diverse set of pages due to the large number of pages; therefore, a team of expert librarians was consulted to help categorize fifty books depending on their fonts. Next, eight books were selected based on the criterion of maximizing font diversity and image quality. Books with low image quality, poor handwriting, ink stains over handwriting, and extremely bad page condition were excluded, leaving these eight books. Recursively, the same process was manually applied to pages within the selected books, to only choose an acceptable five pages from each book. Next, the selected pages were fed to the image processing step to be cropped and enhanced, and then, lastly, the resultant pages were given to specialists to annotate the images as text using text processing software, maintaining the exact order of each line in each page. In other words, we have selected eight books from old Arabic manuscripts after analyzing the whole set of books, and we have used five pages from each book, ensuring high-quality image extraction and correct textual ground truth transcription by Arabic language experts. Now, after that, we organize the dataset book by book. Each book has both the image numbered from 1 to 5 in PNG and the corresponding text in a Word file. This can be followed straightforwardly, as shown in the directory structure in Fig. 1. The books and their authors, orderly, as appearing in the dataset, are the following: Rafaa Al Niqab An Kitab Al Shahab, Al Husain Al Shushawi; Nuzul Al Saereen Ela Allah Rabb Al Aalamin Fee Ahadith Said Al Mursalin, Mahmoud Al Darkazini; Kitab Al Azamah, Ibn Hayyan Al Asbahani; Al Rawd Al Nadheer, Muhammad Mutawalli; Sirr Al Fosoos, Muhammad Abdulbaqi; Al Juzz Alkhamis Min Jamii Al Masanid Wa Al Sunan Al Hadi Li Aqwam Sunan, Ismail Ibn Kathir; Kitab Tareekh Madinat Dimashq Wa Mn Banaha Min Al Mutaqaddimin, Ali Al Rabiy; Dirham Al Suarrah Fee Wad Al Yadain Taht Al Surrah, Muhammad Hashim Al Sindi.

## 4 Experimental Design, Materials and Methods

**Fig. 2:**
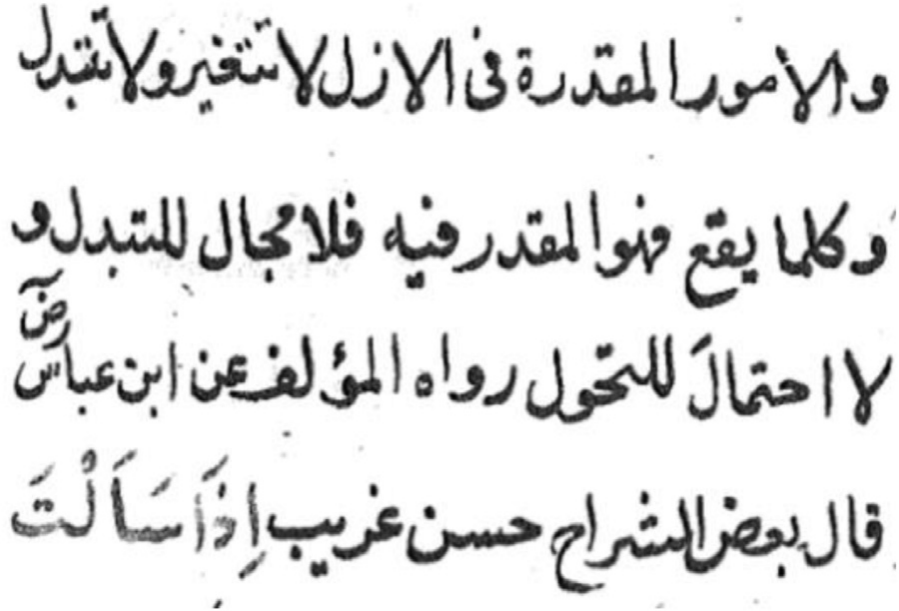
A sample from a single page.

Data Acquisition: The data is collected from the Central Library of the Islamic University of Madinah. The library has manuscript images that are scanned. Eight books out of fifty were selected to annotate and constitute the dataset. Many books were discarded from these fifty books in the first scan due to the low quality they are in or to the repetitive nature of the font. We focused on obtaining manuscripts with the maximum diversity and quality possible. Manuscripts that were of poor quality were excluded. A sample excerpt taken from a single page is shown in Fig. 2.

Data Analysis: A team of expert librarians assisted in cataloging and classifying a wide range of manuscripts, which were eventually placed in 8 buckets, where each bucket contains five images that have high similarity in terms of font. It consists of 640 lines (=29,900 characters).

Data Pre-processing: Image processing software such as Photoshop/GIMP was used to further cut the extra spaces around each page [21].

Data Annotation: Text processing software such as MS Word and Notepad were used to annotate each page. It's important to note that this transcription is exactly matching the page and the lines present in terms of length, which makes it appropriate both at the page and line level.

**Fig. 3:**
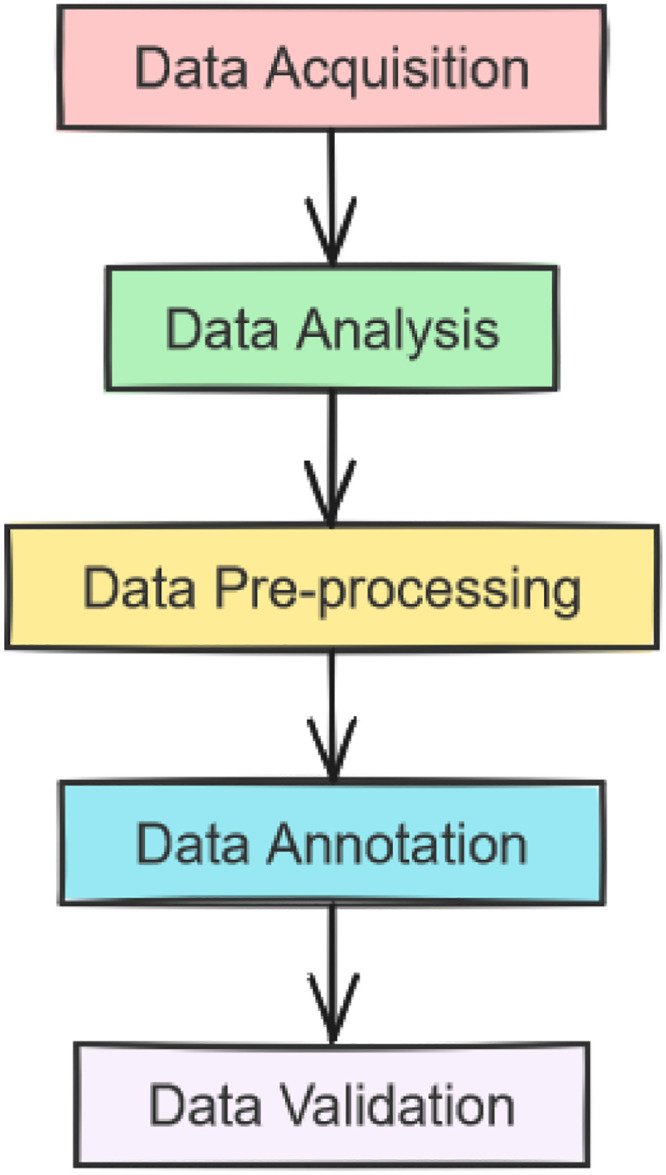
Experimental design.

Data Validation: Manuscript experts and historians assisted in validating the quality of the annotation. This includes ensuring that no line has different words from what is in the corresponding manuscript. Moreover, it ensures that characters are correctly and accurately matching the analogous characters in the original manuscript. Fig. 3 illustrates the complete experimental design.

## Limitations

Due to the difficulty of the task of annotation of ancient texts, the time it consumes, and the labor it requires, as well as the constrained time, budget, and staff provided for this work, only 50 pages have been fully analyzed and their ground truth is transcribed. However, this dataset is ideal both as a test set for Ancient Arabic and Arabic OCR models and as an incorporated part of other datasets for Arabic OCR. In addition, augmentation and generation techniques facilitate this size adequately for training purposes. Most importantly, this selected dataset vastly represents diverse eras and geographies that make it, independently of its sufficient size, of significant importance. On the other hand, this dataset can adequately be used for the text correction task models for Ancient Arabic and Arabic text. Moreover, this amount is a great Kickstarter for deep learning OCR and text correction models, provided the scarcity of such data and the availability of vision methods to manipulate and augment the images. In addition, the diversity of the data in terms of timescale makes it of significant importance for ancient OCR models.

## Ethics Statement

The current work does not involve human subjects, animal experiments, or any data collected from social media platforms.

## CRediT Author Statement

**Safiullah Faizullah**: Conceptualization, Project administration, Funding acquisition, Writing- Reviewing and Editing, and Supervision. **Rayyan Najam**: Data curation, Investigation, Methodology, Formal analysis, Validation, Writing- Original draft preparation, Visualization, Resources, and Software.

## Data Availability

Historical Arabic Handwritten Text Recognition Dataset (Original data) (Mendeley Data).
